# Machine Learning and Computer Vision System for Phenotype Data Acquisition and Analysis in Plants

**DOI:** 10.3390/s16050641

**Published:** 2016-05-05

**Authors:** Pedro J. Navarro, Fernando Pérez, Julia Weiss, Marcos Egea-Cortines

**Affiliations:** 1 DSIE, Universidad Politécnica de Cartagena, Campus Muralla del Mar, s/n. Cartagena 30202, Spain; 2 Genética, Instituto de Biotecnología Vegetal, Universidad Politécnica de Cartagena, Cartagena 30202, Spain; fernando.perez8@um.es (F.P.); Julia.weiss@upct.es (J.W.); marcos.egea@upct.es (M.E.-C.)

**Keywords:** computer vision, image segmentation, machine learning, data normalisation, circadian clock

## Abstract

Phenomics is a technology-driven approach with promising future to obtain unbiased data of biological systems. Image acquisition is relatively simple. However data handling and analysis are not as developed compared to the sampling capacities. We present a system based on machine learning (ML) algorithms and computer vision intended to solve the automatic phenotype data analysis in plant material. We developed a growth-chamber able to accommodate species of various sizes. Night image acquisition requires near infrared lightning. For the ML process, we tested three different algorithms: *k*-nearest neighbour (kNN), Naive Bayes Classifier (NBC), and Support Vector Machine. Each ML algorithm was executed with different kernel functions and they were trained with raw data and two types of data normalisation. Different metrics were computed to determine the optimal configuration of the machine learning algorithms. We obtained a performance of 99.31% in kNN for RGB images and a 99.34% in SVM for NIR. Our results show that ML techniques can speed up phenomic data analysis. Furthermore, both RGB and NIR images can be segmented successfully but may require different ML algorithms for segmentation.

## 1. Introduction

The advent of the so-called omics technologies has been a major change in the way experiments are designed and has driven new ways to approach biology. One common aspect to these technology-driven approaches is the continuous decrease in price in order to achieve high throughput. As a result biology has become a field where big data accumulates, and which requires analytical tools [1]. The latest newcomer in the field of automatic sampling is the so-called phenomics. It comprises any tool that will help acquire quantitative data of phenotypes. Plant growth and development can be considered as a combination of a default program that interacts with biotic and abiotic stresses, light and temperature to give external phenotypes. And measuring, not only the outcome or end point, but also kinetics and their changes is becoming increasingly important to understand plants as a whole and become more precise at experimental designs. One of the newest developments is automatic image acquisition [2].

One of the fields where automatic image acquisition has defined its development is circadian clock analysis as promoters driving reporter genes such as luciferase or Green Fluorescent Protein allowed the identification of mutants and further characterization of the gene network at the transcriptional level [3,4]. Artificial vision systems have been used to study different aspects of plant growth and development such as root development [5], leaf growth [6], flowers and shoots [7] or seedling [8]. An important challenge of image acquisition in plant biology is the signalling effect of different light wavelengths including blue light, red and far red. As a result image acquisition in the dark requires infrared lightning [7,9].

Phenotyping of small plants such as *Arabidopsis thaliana* can be performed with a vertical camera taking pictures of rosettes at time intervals [10]. Larger plants or the parallel phenotyping of several traits require image acquisition from lateral positions [11]. Thus obtaining lateral images or the reconstruction of 3-dimensional images is performed by combination of cameras or moving them to acquire images [12].

Although hardware development requires a multidisciplinary approach, the bottleneck lies in image analysis. Ideally images should be analysed in an automatic fashion. The number of images to be processed when screening populations or studying kinetics can easily go into the thousands. The partition of digital images into segments, known as segmentation is a basic process allowing the acquisition of quantitative data that may be a number of pixels of a bidimensional field, determining the boundaries of interest in an object [13]. Segmentation discriminates between background and defines the region under study and is the basis for further data acquision.

The development of artificial intelligence processes based on machine learning (ML) has been an important step in the development of software for omic analysis and modelling [14]. Examples include support vector machines (SVM) for Illumina base calling [15], *k*-nearest neighbour (kNN) classification for protein localization [16] or Naïve Bayes Classifiers for phylogenetic reconstructions [17]. Furthermore, ML approaches have been used extensively in image analysis applied to plant biology and agriculture [18,19].

Plant growth occurs in a gated manner *i.e.*, it has a major peak during the late night in hypocotyls, stems or large leaves [11,20,21]. This is the result of circadian clock regulation of genes involved in auxin and gibberellin signalling and cell expansion [22]. One of the inputs to the circadian clock is blue light transmitted through proteins that act as receptors such as ZEITLUPE/FLAVIN-BINDING, KELCH REPEAT, F-BOX and LOV KELCH PROTEIN2 [23,24]. Phytochromes absorb red and far red light such as PHYTOCHROME A [25,26]. As a result night image acquisition has to be done with near infrared (NIR) light giving the so-called extended night signal [27]. The aim of this work was to develop the corresponding algorithms to obtain data from day and night imaging. We used machine learning to analyse a set of images taken from different species during day and night. We used the aforementioned SVM, NBC and kNN to obtain image segmentations. Our results demonstrate that ML has great potential to tackle complex problems of image segmentation.

## 2. Materials and Methods

**Figure 1:**
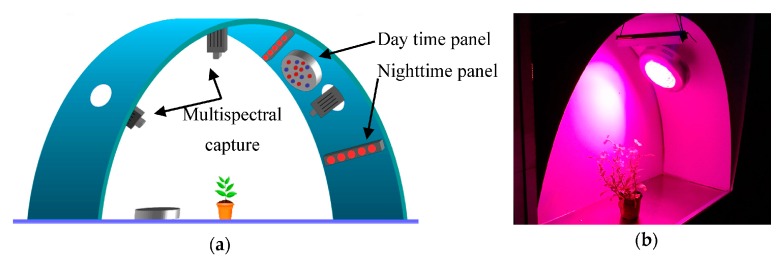
Growth chamber: (**a**) Functional schematic of the system; (**b**) Experiment with *Petunia x hybrida* during daytime in the real system.

Figure 1a shows a schematic of the system. The data acquisition system is composed of four modules which we describe below.

### 2.1. Ilumination Subsystem

We pursued two goals with the illumination subsystem. First we wanted to grow plants under conditions close to their natural environments and second we wanted to acquire pictures during the night-time without interfering with the behaviour of the plant. For this purpose, we have established two illumination periods: daytime and night-time. The illumination subsystem is composed of two LED (light-emitting diode) panels which, allows to carry-out the capture image process and the same time it allows to supply the precise combination of the wavelengths for growing up correctly.

**Figure 2:**
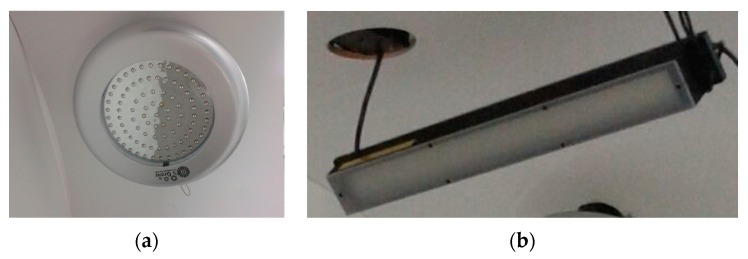
Illumination subsystem (**a**) Daytime LED panel; (**b**) Nightime LED panel.

The daytime LED panel is formed by a combination of five types of LEDs emitting wavelengths with peaks in UV light (290 nm), blue light (450 and 460 nm) and red light (630 and 660 nm). The LED panel has a power of fifty watts. It is usually used for indoor growing of crop plants. The merging of wavelengths produces an illumination with a pink-red appearance (Figure 2a).

The night-time LED panel is composed by a bar of 132 NIR LEDs (three rows of forty four LEDs) with a wavelength of 850 nm (Figure 2b). 

We programmed a system that would give a day/night timing whereby day light was created by turning on the daytime LED. In order to capture night images, the night-time LED panel was turned on for a period between 3 and 5 s coupled to an image capture trigger. The system can be programmed by the user for different periods of day and night lengths and time course of picture acquisition. The minimal period is one picture every 6 s and the maximal is one picture in 24 h.

### 2.2. Capture Subsystem

**Figure 3:**
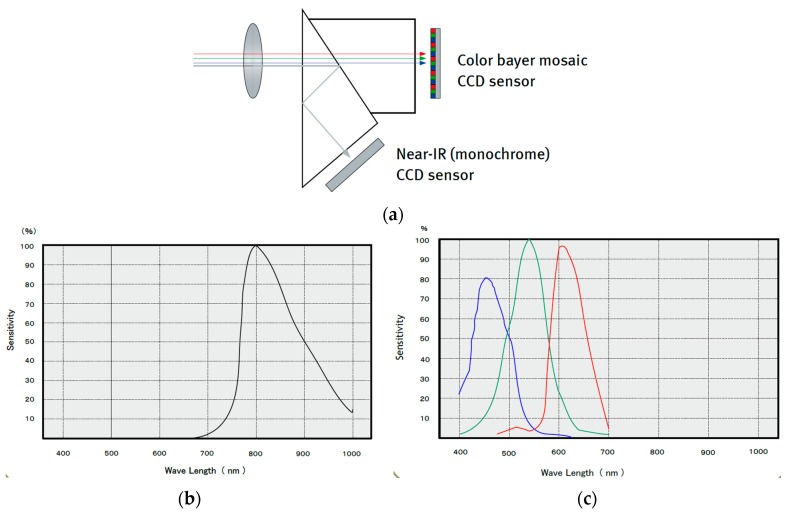
Capture subsystem (**a**) Prism between lens and CCDs; (**b**) Camera NIR-IR response; (**c**) Camera RGB response.

The capture module is in charge of image capture during day and night and the control of the illumination subsystem. The main capture subsystem element is a multispectral 2-channel Charge-Coupled Device (CCD) camera. A prism placed in the same optical path between the lens and CCDs allows a simultaneous capture the visible (or RGB) and NIR image (see Figure 3a). This feature has reduced the amount of cameras being used by the system and has avoided the construction of a mechanical system to move the lenses or the cameras in front of the plants. The camera has a resolution of 1024 (h) × 768 (v) active pixels per channel. During day and night a resolution of 8 bit per pixel was used in all the channels (R-G-B-NIR). Figure 3b,c shows the response of the NIR-CCD and RGB-CCD of the multispectral camera.

**Figure 4:**
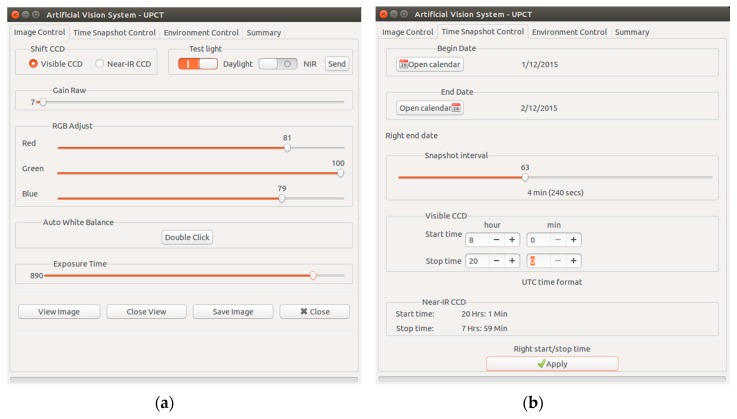
Graphical User Interface for capture subsystem: (**a**) Image control tap; (**b**) Time control tab.

**Figure 5:**
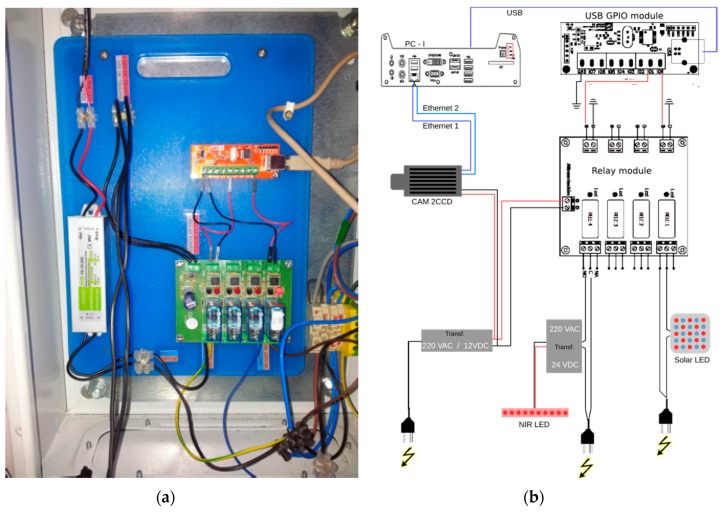
Hardware of the capture subsystem. (**a**) USB-GPIO module (red-board) and opto-coupler relay module (green-board); (**b**) Electric connections between all hardware modules in the growth-chamber.

Capture and illumination subsystems are controlled via a GUI developed in C/C++ (Figure 4a,b). It comprises eight digital input/output channels and six analog ones in an USB-GPIO module (Figure 5a). The system had 10 bit resolution. It was configured using the *termios* Linux library in C/C++.

The second component was the optocoupler relay module. It had four optocoupled outputs, optocoupled to a relay triggering at voltages between 3 and 24 V. Both day light and night light LEDs were connected to two relays (Figure 5b), in such a way that the configuration via the control software dictates the beginning of image acquisition, triggers light turning on or off coordinating the light pulses with the camera during the day and night.

### 2.3. Image Processing Module

Each experiment generates two types of images: one NIR image during the night-time and another RGB image during the daytime. In order to obtain an automatic image segmentation, we designed an algorithm to classify the objects from the images of the experiment in two groups: organs and background. The algorithm developed is divided in three stages.

#### 2.3.1. Extraction of Samples of Images Representative from the Different Classes

During the first stage of the algorithm we have selected a set of representative samples formed by n matrix with size of *k* × *k* pixels of each class. The size of the regions can be of 1 × 1, 16 × 16, 32 × 32, 64 × 64 or 128 × 128 pixels. This will depend of size and morphology of the organ to be classified and of the period of the daytime or night-time involved. During the day we used a single pixel per channel while we used the larger pixel regions to increase the information obtained in the IR channel.

#### 2.3.2. Features Vector

The feature vector is usually composed by a wide variety of different types of features. The most utilized features are related to: intensity of image pixels [28], geometries [29] and textures (first and second-order statistical features) [30,31]. In addition the feature vector is computed over image transformations such as Fourier, Gabor, and Wavelet [32]. Colour images comprise three channels for R, G and B. As a result the amount of information is multiplied by three and the number of possible combinations and image transformations are incremented.

We have applied two types of features vector techniques depending whether the image was captured during daytime (RGB image) or night-time (NIR images). We tested several colour spaces to construct the features vector of daytime: RGB primary space, HSV perceptual space and CIE L*a*b* luminance-chrominance space [33]. The use of a single colour space produced poor results. In order to improve the performance we increased the number of feature vectors. We used cross combinations of the RGB, CIE L*a*b*, HSV and found that the best performance was with RGB and CIE L*a*b*. Thus we constructed the features vector formed by the pixel the corresponding pixel values. In the NIR images, we used a features vector computed over two decomposition levels of the Haar wavelet transform.

##### Colour Images

**Figure 6:**
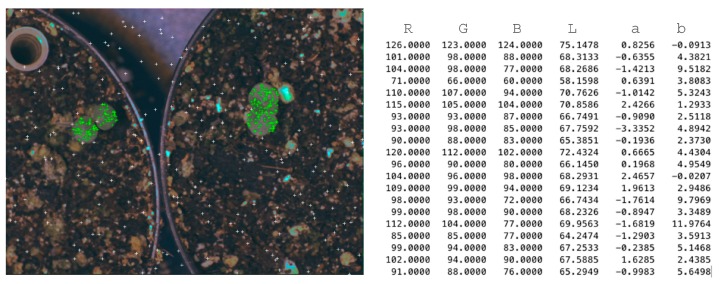
Colour images features vector construction. A matrix of different pixel values corresponding to R, G, B, and L*a*b* colour spaces.

The features vector of the colour images is composed of six elements extracted from the pixel values of two colour spaces: RGB and CIE L*a*b*. We selected a large set of random pixels of each class to construct the features vector Figure 6a (organs-class1-green and background-class2-white). It was necessary to convert RGB colour space of the original image to CIE L*a*b* colour space. Figure 6b shows the twenty values of the features vector of class 1.

##### NIR Images

**Figure 7:**
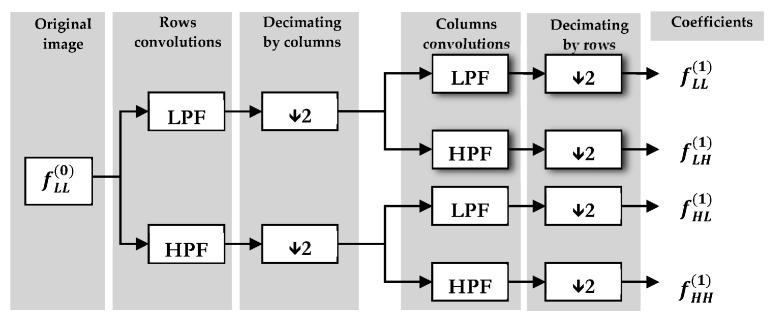
First level of direct 2D-DWT decomposition.

Discrete Wavelet Transformation (DWT) generates a set of values formed by the “wavelet coefficients”. Being *f*(*x*, *y*) an image of *M* × *N* size, each level of wavelet decomposition is formed by the convolution of the image *f*(*x*, *y*) with two filters: a low-pass filter (LPF) and a high-pass filter (HPF). The different combinations of these filters result in four images here described as LL, LH, HL and HH. In the first decomposition level four subimages or bands are produced: one smooth image, also called approximation, $f_{\text{LL}}^{\left(1\right)}\left(x,y\right)$, that represents an approximation of the original image *f*(*x*, *y*) and three detail subimages $f_{\text{LH}}^{\left(1\right)}\left(x,y\right)$, $f_{\text{HL}}^{\left(1\right)}\left(x,y\right)$ and $f_{\text{HH}}^{\left(1\right)}\left(x,y\right)$, which represent the horizontal, vertical and diagonal details respectively. There are several wavelet mother functions that can be employed, like Haar, Daubechies, Coiflet, Meyer, Morlet, and Bior, depending on the specific problem to be identified [34,35]. Figure 7 shows the pyramid algorithm of wavelet transform in the first decomposition level.

In this work we have computed a features vector based on the wavelet transform with basis Haar [36]. The features vector is formed of four elements: maximum, minimum, mean and Shannon entropy of coefficients wavelets calculated in the horizontal, vertical and diagonal subimages in two decomposition levels (see Equations (1)–(5)): We have eliminated the approximation subimage due to it contains a representation decimated of the original image:

(1) $$f_{1,\;..,\;6}=\text{max}\{f_{\text{LH}}^{\left(l\right)}\left(x,y\right),\;f_{\text{LH}}^{\left(l\right)}\left(x,y\right),f_{\text{LH}}^{\left(l\right)}\left(x,y\right)\}\;,\forall l=1,2$$

(2) $$f_{7,..,12}=\text{min}\{f_{\text{LH}}^{\left(l\right)}\left(x,y\right),\;f_{\text{LH}}^{\left(l\right)}\left(x,y\right),f_{\text{LH}}^{\left(l\right)}\left(x,y\right)\},\;\forall l=1,2$$

(3) $$f_{13,..,18}=\text{mean}\{f_{\text{LH}}^{\left(l\right)}\left(x,y\right),\;f_{\text{LH}}^{\left(l\right)}\left(x,y\right),f_{\text{LH}}^{\left(l\right)}\left(x,y\right)\},\;\forall l=1,2$$

(4) $$f_{19,\;..,24}=\text{shannon}\_\text{entropy}\{f_{\text{LH}}^{\left(l\right)}\left(x,y\right),\;f_{\text{LH}}^{\left(l\right)}\left(x,y\right),f_{\text{LH}}^{\left(l\right)}\left(x,y\right)\}\;,\forall l=1,2$$

Shannon Entropy is calculated as the Equation (5):

(5) $$\text{shannon}\_\text{entropy}\left(f_{s}^{\left(l\right)}\right)=-\overset{M/2^{l}}{\underset{i=1}{\sum }}\overset{N/2^{l}}{\underset{j=1}{\sum }}p\left(w_{ij}\right)\;log_{2}\left(p\left(w_{ij}\right)\right)$$

The letter *l* represents the value of the wavelet decomposition level, *s* the subimages (LL, HL, LH, HH) created in the wavelet decomposition, and $w_{ij}$ represents the wavelet coefficient (*i*, *j*), located in the *s*-subimage, at *l*-decomposition level. *p* represents the occurrence probability of the wavelet coefficient $w_{ij}$.

Feature vector has been obtained applying the Equations (1)–(5) to each region in two wavelet decomposition levels with Haar basis. The result was a feature vector of twenty-four elements ($f_{1,..,24}$) per region of size *k* × *k*.

#### 2.3.3. Classification Process

We have tested three machine-learning algorithms: (1) *k*-nearest neighbour (kNN); (2) naive Bayes classifier (NBC), and Support Vector Machine (SVM). The algorithms selected belong to the type of supervised classification. These type of algorithms require of a training stage before performing the classification process.

kNN classifier is a non-parametric method for classifying objects in a multi-dimensional space. After being trained, kNN assigns a specific class to a new object depending on the majority of votes from its neighbours. This measure is based in metrics such as Euclidean, Hamming or Mahalanobis distances. In the implementation of kNN algorithm it is necessary to assign an integer value to *k*. This parameter represents the *k*-neighbors used to carry-out the voting classification. A *k* optimal determination will allow that the good model adjusts to future data [37]. It is recommendable to use data normalisation coupled to kNN classifiers in order to avoid the predominance of big values over small values in the features vector.

NBC uses a probabilistic learning classification. Classifiers based on Bayesian methods utilize training data to calculate an observed probability of each class based on feature values. When the classifier is used later on unlabeled data, it uses the observed probabilities to predict the most likely class for the new features. As NBC works with probabilities it does not need data normalization.

SVM is a supervised learning algorithm where given labeled training data, it outputs a boundary which divides data by categories and categorizes new examples. The goal of a SVM is to create a boundary, called hyperplane, which leads to homogeneous partitions of data on either side. SVMs can also be extended to problems were the data are not linearly separable. SVMs can be adapted for use with nearly any type of learning task, including both classification and numeric prediction. SVM classifier tend to perform better after data normalisation.

In this work we have used raw data and two types of normalisation procedures which have been computed over features space: dn0: without normalisation; dn1: mean and standard-deviation normalization and dn2: mode and standard-deviation normalisation. Features space of each class is composed by a *m* × *n* matrix (see Equation (6)):

(6) $$f_{ij}^{nC}=\left[\begin{array}{c} \begin{array}{ccc} f_{11}^{1} & \cdots & f_{1n}^{1}\\ \vdots & \ddots & \vdots \\ f_{1m}^{1} & \cdots & f_{mn}^{1} \end{array}\\ \begin{array}{c} \cdot \\ \cdot \\ \cdot \\ \begin{array}{ccc} f_{11}^{nC} & \cdots & f_{1n}^{nC}\\ \vdots & \ddots & \vdots \\ f_{1m}^{nC} & \cdots & f_{mn}^{nC} \end{array} \end{array} \end{array}\right]\nabla \;i=1,\ldots ,m\;;\;j=1,\ldots ,n\;;nC=1,\;2$$

Being *i*-th row, the vector of features *i*-th of features space formed by *n* features. *m* represents the number of vectors in the features space and *nC* represents the number of classes in th space.

The normalised features space, $F_{ij}^{nc}$, depending on the normalisation types (dn0, dn1, dn2) is computed as is shown in the Equation (7):

(7) $$F_{ij}^{nc}=\left[\begin{array}{ccc} \frac{f_{11}^{1}-st_{1}}{st_{2}} & \cdots & \frac{f_{1n}^{1}-st_{1}}{st_{2}}\\ \vdots & \ddots & \vdots \\ \frac{f_{1m}^{1}-st_{1}}{st_{2}} & \cdots & \frac{f_{mn}^{1}-st_{1}}{st_{2}}\\ & \begin{array}{l} \cdot \\ \cdot \\ \cdot \end{array} & \\ \frac{f_{11}^{nC}-st_{1}}{st_{2}} & \cdots & \frac{f_{1n}^{nC}-st_{1}}{st_{2}}\\ \vdots & \ddots & \vdots \\ \frac{f_{1m}^{nC}-st_{1}}{st_{2}} & \cdots & \frac{f_{mn}^{nC}-st_{1}}{st_{2}} \end{array}\right]\to \{\begin{array}{c} dn0\{\begin{array}{c} st_{1}=0\\ st_{2}=1 \end{array}\begin{array}{cc} raw & data \end{array}\\ dn1\{\begin{array}{c} st_{1}=mean(f_{ij}^{nC})\\ st_{2}=StdDes(f_{ij}^{nC}) \end{array}\\ dn2\{\begin{array}{c} st_{1}=mode(f_{ij}^{nC})\\ st_{2}=StdDes(f_{ij}^{nC}) \end{array} \end{array}$$

To obtain the best result in classification process, the ML algorithms were tested with different configuration parameters. kNN was tested with Euclidean and Minkowski distances with three type of data normalisation, NBC was tested with Gauss and Kernel Smoothing Functions (KSF) without data normalisation, and SVM was tested with linear and quadratic functions, on three types of data normalisation. The ML algorithms used two classes of objects. One for the plant organs and a second one for the background. In all of them we applied the leave-out cross validation (LOOCV) method to measure of the error of the classifier. Basically LOOCV method extracts a sample of the training set and it constructs the classifier with the remaining of the training samples. Then it evaluates the classification error and the process is repeated for all the training samples. At end the LOOCV method computes the mean of the errors and it obtains a measure of how model is adjusted to data. This method allows comparing the results of the different ML algorithms, provided that they will be applied to same sample data. Table 1 shows a summary of parameters used for the classification process.

### 2.4. Experimental Validation

In order to test and validate the functioning of the system and ML methods, acquired pictures of *Antirrhinum majus* and *Antirrhinum linkianum* were used to analyse growth kinetics. The camera was positioned above the plants under study. The daylight LED panel was above the plants while the night-time LED was at a 45°. Data acquisition was performed for a total of six days. We obtained one image every 10 min during day and night. Day night cycles were set to 12:12 h and triggering of the NIR LED for image acquisition during the night was done for a total of 6 s.

In the experiment we obtained 864 colour images from the RGB sensor which were transformed to CIE L*a*b* colour space and 864 gray scale images from the NIR sensor. From each group (RGB and NIR images) we obtained fifty ground-truth images which were segmented manually by human experts. From the fifty ground-truth colour images, we selected 1200 samples of 1 pixel, which we used to train the RGB image processing ML algorithms. From the second fifty ground-truth NIR images we took 1200 regions of 32 × 32 pixels which were to train the NIR image processing ML algorithms. In both cases we selected 600 samples belonging to organs class and 600 samples belonging to background class.

**Figure 8:**
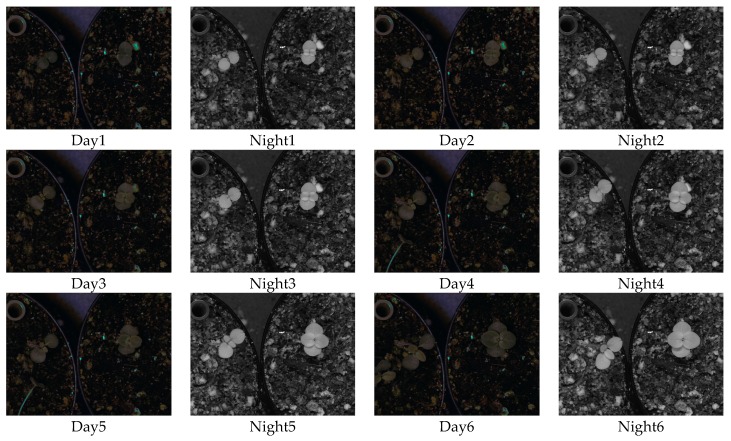
Images of the experiment captured every 12 h.

Figure 8 shows two images from each day of the experiment for different capture periods. We can distinguish easily the growth stages of the two species during the daytime and night-time.

## 3. Results and Discussion

We evaluated the results of training stage of the ML algorithms with a leave-one-out cross-validation method (LOOCV) and with the Receiver Operating Characteristic (ROC) curve over data training sets obtained from RGB and NIR images. LOOCV and ROC curves have been applied under the different ML configurations shown in the Table 1. This allowed to select the optimal ML algorithm to be applied to each type of image depending on when it was captured: during daytime or night-time. Once we determined the optimal ML algorithm for each image set, we used the metric miss-classification [37] to evaluate the final performance of the implemented ML algorithms.

### 3.1. LOOCV

Table 2 shown the errors obtained after to apply LOOCV to the two images groups respectively. In both images groups the minimum error in data model adjust is produced with kNN classifier. Data normalisation based on in the mean (dn1) produced the best result in both cases, too. The maximum error is produced by the NBC classifier, with KSF and Gauss kernel, respectively.

### 3.2. ROC Curves

**Figure 9:**
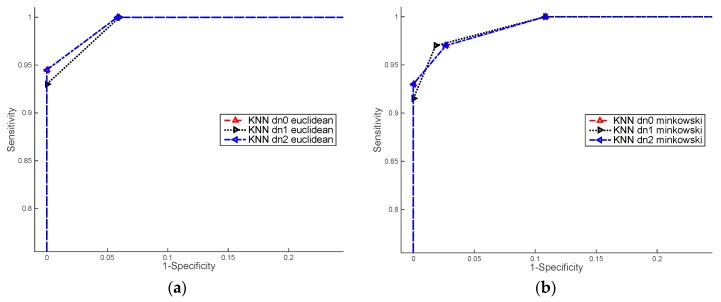

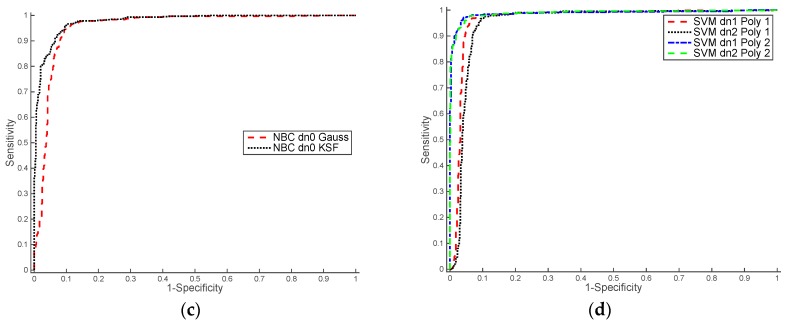
ROC results for training colour images. (**a**) kNN classifier with distance Euclidean and data normalisation: dn0, dn1 and dn2; (**b**) kNN classifier with distance Minkowski and data normalisation: dn0, dn1 and dn2; (**c**) BN classifier with Gauss and KSF kernels and data normalisation dn0; (**d**) SVM classifier with lineal and quadratic polynomial functions and data normalisation: dn1 and dn2.

We evaluated the performance of the ML algorithms using Receiver Operating Characteristic (ROC) curve. The ROC curve is created by comparing the sensitivity (the rate of true positives TP, see Equation (8)), *versus* 1-specificity (the rate of false positives FP see Equation (9)), at various threshold levels [38]. The ROC curves, shown in Figure 8 and Figure 9, allow comparing the results between of ML algorithms per each groups of images:

(8) $$Sensitivity=\frac{TP}{TP+FN}$$

(9) $$Specificity=\frac{TN}{TN+FP}$$

The Area Under the Curve (AUC) is usually used by ML to compare statistical models. AUC can be interpreted as the probability that the classifier will assign a higher score to a randomly chosen positive example than to a randomly chosen negative example [39,40].

Figure 9 shows ROC curves computed over the set of colour images training. The higher values of AUC were obtained by kNN classifier with Euclidean distance. Concerning the data normalisation, we achieved similar results with raw data and normalisation based on the mode and standard deviation (dn0 and dn1).

**Figure 10:**
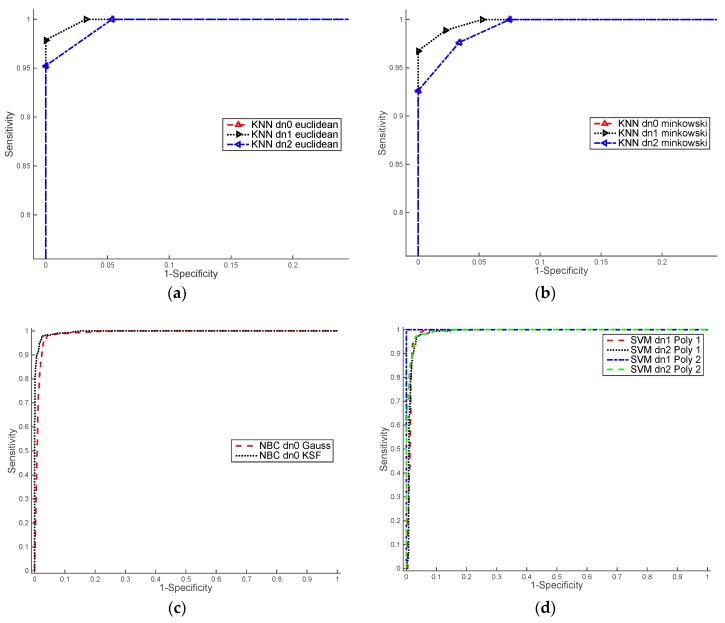
ROC results for training NIR images. (**a**) kNN classifier with distance Euclidean and data normalisation: dn0, dn1 and dn2; (**b**) kNN classifier with distance Minkowski and data normalisation: dn0, dn1 and dn2; (**c**) BN classifier with Gauss and KSF kernels and data normalisation dn0; (**d**) SVM classifier with lineal and quadratic polynomial functions and data normalisation: dn1 and dn2.

Figure 10 shows ROC curves computed over the set of NIR images training. We can observe that the best results were obtained with the SVM classifier with quadratic functions and using a normalised data based on the mean and standard deviation (dn1). Table 3 shows AUC values obtained from ROC curves of the Figure 9 and Figure 10.

**Figure 11:**
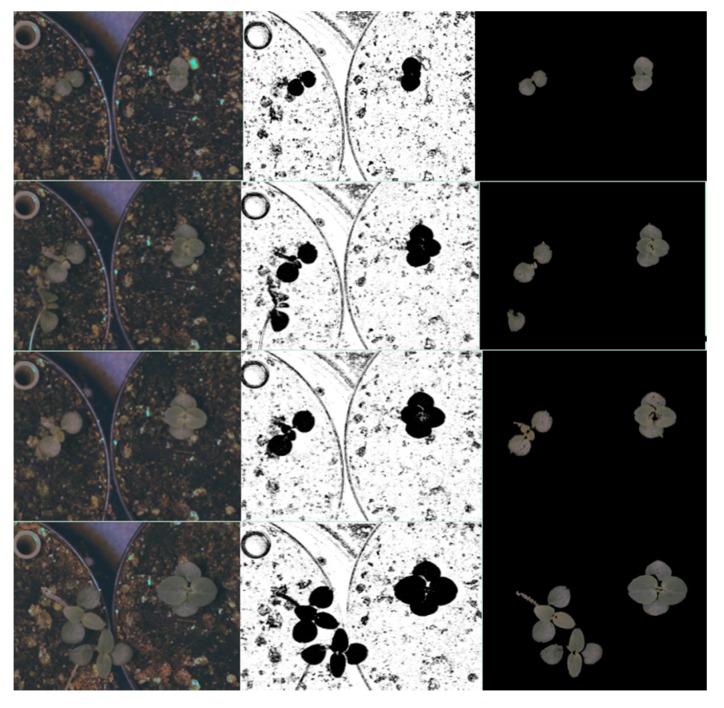
Results of the colour image segmentation based on kNN classifier in different growing stages. Left shows four colour images selected at different growth stages. The second column presents the result of segmentation using kNN classifier with Euclidean distance and without normalisation data (dn0). The third column shows the results after post-processing stage.

**Figure 12:**
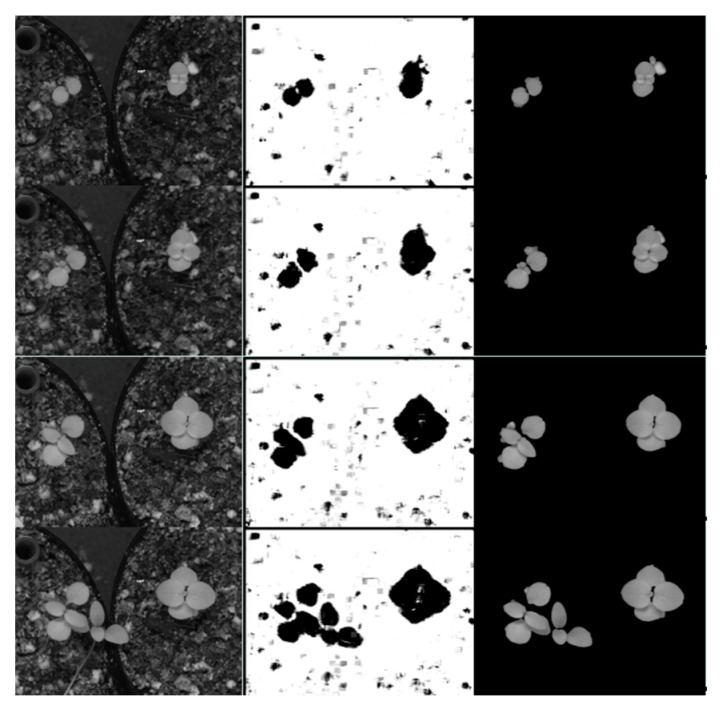
Results of the NIR image segmentation based on SVM. Left shows four NIR images chosen at different growth stages growing during night-time. The second column shows the segmentation results after application of the SVM classifier with quadratic function and with data normalisation dn1. The third column shows the results after post-processing stage.

In both cases after classification stages, we performed a postprocessing stage composed of morphological operations and an area filter to eliminate noise and small particles. The segmented images were merged with the original images (Figure 11 and Figure 12).

### 3.3. Error Segmentation

The misclassification error (ME) represents the percentage of the background pixels that are incorrectly allocated to the object (*i.e.*, to the foreground) or *vice versa*. The error can be calculated by means of Equation (10), where *B_GT_* (Background Ground-Truth image) and *O_GT_* (Object Ground-Truth image) represent the ground-truth image of the background and of the object taken as reference, and *B_T_* (Background Test) and *O_T_* (Object Test) represent the image to be assessed. In the event that the test image coincides with the pattern image, the classification error will be zero and therefore the performance of the segmentation will be the maximum [41].

(10) $$ME=\frac{\left|B_{GT}\cap^{\text{​}}B_{T}\right|+\left|O_{GT}\cap^{\text{​}}O_{T}\right|}{\left|B_{GT}\right|+\left|O_{GT}\right|}$$

The performance of the implemented algorithms is assessed according to the Equation (11):

(11) η=100·(1−ME)

Table 4 shows mean values computed after to segment the fifty ground-truth images of each group with optimal ML algorithm (kNN and SVM) selected in the previous subsection.

The performance of the image segmentation calculated shows excellent results in both groups (Table 4). It has been necessary to increase the complexity of the features vector in the NIR images (using Wavelet transform) to obtain a similar results of performance. This is probably an expected result as RGB images had three times more of information than a NIR image.

## 4. Conclusions and Future Work

In this work we have developed a system based on ML algorithms and computer vision intended to solve the automatic phenotype data analysis. The system is composed by a growth-chamber with capacities to perform experiments with numerous species. The design of the growth-chamber has allowed easy positioning of different cameras and illuminations. The system can take thousands of images through the capture subsystem, capture spectral images and it creates time-lapse series of the specie during the experiment. One of the main goals of this work has been to capture images during the night-time without affecting plant growth.

We have used three different ML algorithms for image segmentation: *k*-nearest neighbour (kNN), Naive Bayes classifier (NBC), and Support Vector Machine. Each ML algorithm was executed with different kernel functions: kNN with Euclidean and Minkowski distances, NBC with Gauss and KSF functions and SVM with linear and quadratic functions. Furthermore ML algorithms have been trained with two types of data normalisation: dn0 (raw data), dn1 (mean and standard deviation) and dn2 (mode & standard deviation). Our results show that RGB images are better classified with the kNN classifier, Euclidean distance and without data normalisation. In contrast, NIR images performed better with SVM classifier with quadratic function and with data normalisation dn1.

In the last stage we have applied ME metrics to measure the image segmentation performance. We have achieved a performance of 99.3% in both ground-truth colour images and ground-truth NIR images. Currently the algorithms are being used in an automatic image segmentation processing to study circadian rhythm in wild type lines, transposon-tagged mutants and transgenic lines with modifications in genes involved in the control of growth and the circadian clock. Regarding future work, we consider important to identify a new feature vector which produces better performance rates, improve the illumination subsystem, reduce the computation time of the windowing segmentation in NIR images.

## Figures and Tables

**Table 1:** kNN, NBC and SVM configuration parameters.

Configuration	kNN	NBC	SVM
**method**	Euclidean, Minkowski	Gauss, KSF	Linear, quadratic
**data normalisation**	dn0, dn1, dn2	dn0	dn1, dn2
**metrics**	LOOCV, ROC	LOOCV, ROC	LOOCV, ROC
**classes**	2	2	2

**Table 2:** Colour images and NIR images. LOOCV error for kNN, NBC, SVM.

	Classifier	kNN		NBC		SVM	
	*Configuration*	*Euclidean*	*Minkowski*	*Gauss*	*KSF*	*Linear*	*Quadratic*
**Colour**	**dn0**	0.0283	0.0433	0.0750	0.0758	-	-
	**dn1**	**0.0242**	0.0467	-	-	0.0533	0.0383
	**dn2**	0.0283	0.0433	-	-	0.0667	0.0450
**NIR**	**dn0**	0.0288	0.0394,	0.0356	0.0319	-	-
	**dn1**	**0.0169**	0.0281	-	-	0.0326	0.0319
	**dn2**	0.0288	0.0394	-	-	0.0344	0.0325

**Table 3:** Colour images and NIR images. AUC for kNN, NBC, SVM.

	Classifier	kNN		NBC		SVM	
	*Configuration*	*Euclidean*	*Minkowski*	*Gauss*	*KSF*	*Linear*	*Quadratic*
**Colour**	**dn0**	**0.9984**	0.9974	0.9542	0.9778	-	-
	**dn1**	0.9979	0.9976	-	-	0.9622	0.9875
	**dn2**	**0.9984**	0.9974	-	-	0.9496	0.9886
**NIR**	**dn0**	0.9987	0.9979	0.9877	0.9963	-	-
	**dn1**	0.9975	0.9993	-	-	0.9867	**1.000**
	**dn2**	0.9987	0.9979	-	-	0.9868	0.9932

**Table 4:** Performance in the image segmentation.

Classifier	kNN	SVM
**Performance**	99.311%	99.342%

## Abbreviations

The following abbreviations are used in this manuscript:

LM	Machine learning
KSF	Kernel smoothing function
kNN	*k*-nearest neighbour
NBC	Naïve Bayes classifier
SVM	Support vector machines
LOOCV	Leave-one-out cross validation method
AUC	Area under curve
ME	Misclassification error
